# Public Stigma towards Older Adults with Depression: Findings from the São Paulo-Manaus Elderly in Primary Care Study

**DOI:** 10.1371/journal.pone.0157719

**Published:** 2016-06-28

**Authors:** Marcia Scazufca, Maria Clara P. de Paula Couto, Hsiang Huang, Rachel Kester, Patrícia Emília Braga, Érica T. P. Peluso, Sérgio L. Blay, Paulo R. Menezes, Euler E. Ribeiro

**Affiliations:** 1 Laboratory of Psychopathology and Psychiatric Therapeutics (LIM-23), Institute of Psychiatry, University of São Paulo, São Paulo, Brazil; 2 Department of Psychiatry, Cambridge Health Alliance, Cambridge, Massachusetts, United States of America; 3 Department of Preventive Medicine, Faculty of Medicine, University of São Paulo, São Paulo, Brazil; 4 Anhanguera University of São Paulo (UNIAN), São Paulo, Brazil; 5 Department of Psychiatry, Federal University of São Paulo, São Paulo, Brazil; 6 Open University of the Third Age, Amazonas State University, Manaus, Amazonas, Brazil; University of Stellenbosch, SOUTH AFRICA

## Abstract

This study investigates three domains of public stigma (perceived negative reactions, perceived discrimination, and dangerousness) against older adults with depression. The sample comprised of older adults registered with primary care clinics (n = 1,291) and primary health care professionals (n = 469) from São Paulo and Manaus, Brazil. Participants read a vignette describing a 70-year-old individual (Mary or John) with a depressive disorder and answered questions measuring stigma. The prevalence of the three stigma domains was between 30.2 and 37.6% among older participants from São Paulo and between 27.6 and 35.4% among older participants from Manaus. Older adults from both cities reported similar prevalence of perceived stigma. Key factors associated with stigmatizing beliefs among older participants were reporting depressive symptoms, having physical limitations, and identifying the case of the vignette as a case of mental disorder. Among health professionals, the prevalence of the three stigma domains was between 19.8 and 34.8% in São Paulo and 30.2 and 44.6% in Manaus. The key factor associated with stigma among primary health care professionals was city, with consistently higher risk in Manaus than in São Paulo. Findings confirm that public stigma against older adults in Brazil is common. It is important to educate the public and primary health care providers in Brazil on stigma related to mental illness in order to reduce barriers to adequate mental health treatment.

## Introduction

Depression is a common chronic condition among older adults[1] and is associated with poor quality of life, disability, and increased health care utilization and cost [2–4]. Brazil is a middle-income country undergoing a sociodemographic transition, with one of the fastest ageing populations in the world. According to the latest national census, among the 190 million Brazilian inhabitants, 7.4% (i.e., 14 million) are 65 years and older [5]. By 2050, the population of those aged above 65 years old is projected to grow from 14 million in 2010 to approximately 65 million [6]. Given this projected increase in the aged population and the associated chronic conditions including depression, effective treatment for those with depression is critically important.

Barriers exist that prevent older adults with depression from receiving adequate treatment including under-recognition, under-treatment, difficulties in making appointments, and negative attitudes/stigma against depression [7]. Self-stigma and public stigma towards depression may prevent individuals from seeking mental health treatment [8]. Among older patients with major depression, perceived stigma predicts early treatment discontinuation [9]. Adding to the stigma attached to mental illness, prejudice against older adults (i.e., ageism) may lead these individuals to be doubly stigmatized due to older age and depression [10]. In Brazil, studies provide evidence in favor of a predominantly negative social representation of older adults and the aging process with a focus on physical limitations, illness, cognitive and physical decline, loneliness, poverty, and incompetence [11–13].

Public stigma is defined as negative beliefs about a group or stereotypes held by the general public[14]. In a Brazilian study that investigated public stigma towards depression in a community sample, when presented with a vignette of a 30-year-old individual with depression, 56% of participants reported that the person in the vignette could be potentially dangerous [15]. Furthermore, this individual was perceived as being capable of arousing negative reactions by 49% of the sample, and as capable of arousing discrimination in society by 41%. Importantly, identification of the vignette as a case of mental illness was associated with dangerousness, perceived negative reactions and perceived discrimination. However, in this study, the case in the vignette was a young adult with depression. Additionally, individuals 65 years or over were not included as respondents in the survey. In this regard, studies show that the effects of age on public stigma are conflicting. For example, some studies show that age is positively associated with stigma, with older adults declaring more stigma than younger people [16, 17]. Contrary to that, other studies show that either stigma is higher in young people or that age is unrelated to public stigma [18–20].

Past studies have shown that health care professionals hold negative/stigmatizing attitudes towards those with mental illness [21] and towards those with depression in particular [22, 23]. Notably, to date no previous studies investigated public stigma towards depression in older people among community dwelling older adults. Studies about public stigma towards depression among mental health care professionals have been previously conducted [22], but there is no information about the topic among primary care professionals. Jorm and colleagues [24] investigated attitudes against people with depression among the general public and health care professionals (general practitioners, psychiatrists, and clinical psychologists). To that end, they used a vignette describing a 30-year old individual with depression. Findings showed that the public and the clinical psychologists were less likely to believe there would be discrimination than the general practitioners and psychiatrists. In line with the results presented by Jorm and colleagues in 1999, Nordt and colleagues [22] investigated attitudes of mental health professionals and the general population towards mental illness. The authors assessed stereotypes of mentally ill people, restrictions on mentally ill people (e.g., revocation of the driver´s license), recognition of mental illness (schizophrenia and major depression), and social distance (i.e., unwillingness to interact). Findings revealed that as compared to the general population, mental health professionals had more negative stereotypes against mentally ill people; they accepted less often restrictions toward mentally ill people, were more accurate to recognize cases of mental illness, and showed the same degree of social distance as compared to the general population. The authors concluded that despite the better knowledge of mental health professionals, their attitudes about mental illness are not more positive than in the general population. These two studies revealed that health professionals present more stigmatizing attitudes towards people with mental illnesses than the general public. More recently, Li and colleagues [25] showed that levels of stigma against people with mental illness are high among community mental health staff in China.

Given that there are not enough mental health specialists to meet the needs of those with mental illness on a population level adequately, the World Health Organization (WHO) recommends the consideration of managing depression in primary care in order to increase mental health access [26]. For this reason, we aimed to investigate two groups that are intrinsically important for improving the identification and treatment of older people with depression in primary care: older individuals registered with primary care clinics and primary health care professionals. Beyond investigating their perceptions (stigma) towards depression, we also aimed to study if sex, age, income and place of residence in Brazil (São Paulo and Manaus) affected their negative attitudes/stigma about depression. In addition, we also studied if older people and primary health care professionals that reported physical problems and depression, and health care professionals with different experience at work presented different views about depression than those without these characteristics. Hence, we expect that the results of the present study can be helpful for planning interventions aimed at decreasing public stigma towards depression in society and particularly among primary care professionals.

## Methods

### Study design

This study is part of a larger cross-sectional investigation on the prevalence of depression and associated factors in older adults registered with Primary Care Clinics (PCC) within the Family Health Strategy framework (FHS) in the cities of São Paulo and Manaus. The current investigation examining older adults' and health care professionals' attitudes/perceptions towards elderly individuals with depression is descriptive and thus no *a priori* hypothesis has been formulated.

### Study setting and participants

The Family Health Strategy (FHS) is one of the primary health care models within the public health system in Brazil (i.e., the Brazilian Unified Health System or SUS). The FHS is established throughout the Brazilian territory, mostly in the poorer areas. As of December 2015, there have been 40,162 family health teams (FHTs) implemented across the entire country, covering 123,605,306 Brazilians, corresponding to approximately 60% of the population [27]. The FHTs are based on Primary Health Clinics (PCC). Each FHT consists of one family physician, one nurse, one nursing assistant, and up to 12 community health workers [28]. FHTs provide comprehensive and longitudinal care for up to 4,000 inhabitants from a defined catchment area.

For this study, we have chosen two cities situated in very different regions of Brazil. São Paulo (in Southeastern Brazil) and Manaus (in Northern Brazil) are the capitals of the São Paulo State and the Amazon State, respectively. The distance from São Paulo to Manaus is 3,885 km (2,414 miles). São Paulo is one of the largest cities in the world with approximately 12 million inhabitants and over 1 million aged 60 years or older [5]. It is also the most populated and wealthiest city in Brazil, and is surrounded by other important financial and industrial cities. Manaus has approximately 1,800,000 inhabitants with around 100,000 aged 60 years old or older [5]. Manaus is in the middle of the Amazonian jungle and is the most populated city of the Amazon rainforest. Although Manaus is the most important industrial and financial center in Northern Brazil, it is quite isolated from the rest of the country. For most of the year, the only way to reach Manaus is by air or by boat due to the rainy season.

The samples of older adults in São Paulo and Manaus were selected using three criteria. We first randomly selected PCCs with FHTs in the Northern area of São Paulo and in all of the four health districts of Manaus. In São Paulo, each PCC had up to seven FHTs and in Manaus, each PCC had only one FHT. For this reason, in São Paulo we randomly selected 2–4 FHTs in each PCC. We then asked the manager of each PCC to provide us with a list with all individuals aged 60 years and over registered with each FHT and a list with all health professionals from these same FHT selected to participate in the study. From this list, we randomly selected 20 older individuals from each FHT to participate in the study. The selection criteria for selecting participants were based on age and sex (11 women: 5 aged 60–69 years old, 4 aged 70–79 years old, 2 aged 80 years old or over; 9 men: 4 aged 60–69 years old, 3 aged 70–79 years old, 2 aged 80 years old or over). Individuals who declined to participate were replaced by individuals of the same age and sex included in a reserve list. The final population size of older adults considering the effect of cluster sampling was 18,380.072, design df = 70 (São Paulo = 11,518; Manaus = 6,861).

The samples of primary health care professionals in the two cities were comprised of all professionals working in each of the FHTs (i.e. 6 community health workers, 2 nursing assistants, 1 nurse and 1 family physicians) selected to participate in the study. We asked the manager of each PCC to provide us a list with all health professional from each FHT of the PCC.

### Measures

The outcomes studied with older participants and health care professionals were three domains of public stigma: perceived negative reactions, perceived discrimination, and dangerousness. We presented a vignette describing a 70-year-old individual (Mary or John) with a depressive disorder (according to DSM-IV and ICD-10) to all participants before asking the three questions about public stigma (see Box 1). Response options were “yes” or “no”. The first two questions are related to *perceived stigma* (i.e., an individual's perception of what other people think and feel about depression) while the third question is related to *personal stigma* (i.e., an individual's personal thoughts and beliefs about depression). This vignette has been used in a previous study examining stigma against depression in Brazilian adults [15]. It was developed following the study of Jorm and colleagues [29] and evaluated by three experienced psychiatrists before being used in a study by Peluso and colleagues in Brazil [15]. Because in the present study we were interested in public stigma about older adults with depression, the age of the person in the vignette was modified to 70 years old. To avoid response bias related to gender, the gender of the individual described in the vignette was randomly distributed between interviewees.

**Box 1. Vignette describing a 70-year-old individual (Mary or John) with a depressive disorder.**

“John (Mary) is 70 years old and has been feeling much sadder than usual in recent months almost all the time. He (she) has lost interest in activities he (she) used to like, is irritated and without energy for doing things almost all the time. He (she) has also lost his (her) appetite, weight, and has difficulty sleeping. His (her) relatives have noted that he (she) is discouraged, cries a lot and that he (she) is unable to carry out his (her) chores the way he (she) used to.”

Questions about the three dimensions of public stigma:

1. *Perceived negative reactions*: If people that are close to John/Mary, such as relatives, friends or acquaintances knew what has happened to him/her, do you believe they would have negative ideas about him/her?
2. *Perceived discrimination*: If people that are close to John/Mary, such as relatives, friends or acquaintances knew what has happened to him/her, do you believe they would distance themselves from him/her or avoid getting close to him/her?
3. *Dangerousness*: In your opinion, could a person like John/Mary commit a violent act against other people?

The independent variables for the older participants were sociodemographic characteristics (gender, age, level of education, marital status, and family income), self-reported physical morbidities, physical limitations and major depression. Depression was assessed with the Patient Health Questionnaire [30]. The PHQ-9 has nine items that cover the symptoms for DSM-IV criteria for depression (e.g., “Little interest or pleasure in doing things”, “Felling down, depressed or hopeless”, “Felling tired or having little energy”, etc.). Each item is scored from “0” (not at all) to “3” (nearly every day). In the PHQ-9 validation study [31], a PHQ-9 score≥10 was found to have a sensitivity of 88% and a specificity of 88% for major depression with scores of 5, 10, 15, and 20 indicating mild, moderate, moderately severe, and severe depression, respectively. In Brazil, the PHQ-9 has been validated in a population-based sample of adults [32]. Self-reported morbidities were assessed by asking participants if they had 22 health problems common among elderly people (e.g., hypertension, diabetes, heart and lung problems, eye problems, etc). Whenever the problem was present, participants were asked if the problem negatively affected their lives. Presence of physical morbidities was dichotomised as “yes” (presence of at least one physical problem associated with negative effects) or “no.” Physical limitations were assessed with the 12-item “World Health Organization Disability Assessment Schedule 2.0” (WHO-DAS 2.0), which measures levels of disability according to the definitions of the International Classification of Functioning, Disability and Health (ICF) [33]. Each item is assessed in a Likert scale varying from “0” (no difficulty) to “4” (extreme difficulty). As used in previous studies, the total score was standardized, varying from 0 to 100. The presence of physical limitations was dichotomized as “yes” and “no” according to the WHO-DAS 2.0 90th percentile score. The 90th percentile indicates a category of severe disability. Although this is an arbitrary procedure, it has been recommended to model the scores of the WHO-DAS II as a dichotomous outcome [34, 35].

The independent variables assessed with health care professionals were sociodemographic characteristics (gender, age, marital status, and family income), perception of health status (very good, good, not good), major depression (PHQ-9≥10), professional status at the PCC (community health worker, nursing assistant, nurse, family physician), and if the professional worked with patients with mental health problems at the PCC (“yes” or “no”).

Older participants and health care professionals were asked if they identified the individual presented in the vignette as having a mental disorder ("yes" or "no"). This question was used to assess the accuracy in recognizing mental illness in the vignette. One would expect that better knowledge about mental illness, especially among health care professionals, would lead to positive outcomes, such as initiation of treatment and less stigmatizing attitudes. However, previous studies have shown the contrary, with better knowledge of mental health being associated with more stigma. [22,24]

### Procedures

After obtaining ethical approval from Ethics Committees (see Ethics section) in São Paulo and in Manaus, we contacted the institutions responsible for the management of the PCCs in the two cities. The PCC managers helped with reaching health care professionals and older adults. Health care professionals were interviewed face-to-face at the PCC, and older adults, at their households. Trained interviewers used a tablet to record responses electronically. Participants provided written informed consent before the data were collected. Before any data were analyzed, data were checked for inconsistencies.

### Statistical Analysis

Descriptive analyses were carried out to compare main characteristics of older participants and health care professionals from the two cities. Because all the variables were categorical, chi-squared analysis was used. As the sample of older adults was weighted, we used the Rao-Scott chi-square test, which is a design-adjusted version of the Pearson chi-square test. Prevalence of the three stigma domains and its 95% confidence intervals was estimated for older participants and health professionals. To describe factors associated with each stigma domain, unadjusted Poisson regression models were built and risk ratios (RR) and its 95% confidence intervals (95% CI) were estimated for each independent variable. Variables with p-value ≤ 0.10 in the unadjusted analyses were entered in multivariate Poisson regression models. The final multivariate models present only variables with p-values <0.05. Although binary outcomes are usually analysed with logistic regression, there are some situations in which it is more appropriate to estimate a RR instead of an odds ratio (OR). For example, when the outcome is common (>10%) Poisson regression is recommended [36]. In the present study, the prevalence of the three outcome variables was high among older participants (≥27.6%) and health care professionals (≥19.8%). Therefore, Poisson regression models were performed. For the sample of older adults, the results presented for prevalence and risk ratio estimates considered the effect of the cluster sampling used to select these participants.

The following independent variables were included in the unadjusted analyses for the older participants: city, sex, age, education level (0–4, ≥5 years), marital status (married/partnered, single/separated), family income (R$: up to 1000, 1001–2000, and ≥2001), presence of medical morbidities (“yes” or “no”), presence of physical limitations (“yes” or “no”), depression (PHQ9≥10), and identification of the individual presented in the vignette as a case of mental disorder. The following independent variables were included in the unadjusted analyses for the health care workers: city, sex, age, marital status (married/partnered, single/separated), family income (R$1000–2000, 2001–4000, or ≥ 4001), self-reported health status (very good, good, not good), depression (PHQ9≥10), health care worker type (community health worker, nursing assistant, nurse, family physician), if the professional works with patients with mental disorders, and identification of the vignette as a case of mental disorder. Data were analyzed using STATA version 11.2 (College Station, TX).

## Results

We included 1,291 older adults and 469 primary health care professionals in the study. Thirteen PCCs were included in São Paulo and 34 in Manaus. In São Paulo, the sample comprised 227 primary health care professionals and 619 older adults. In Manaus, the sample comprised 242 primary health care professionals and 672 older adults. The sample of health care professionals corresponds to approximately 70% of those eligible in each center. The main reasons for health care professionals not participating in the study were being on leave from work, and refusal.

Tables 1 and 2 summarize the demographic and clinical characteristics of the older participants and health care professionals, respectively. Overall, older participants had a low level of education (75.7% up to 4 years) and 81.7% had a family income up to 2,000 Brazilian Reais. Prevalence of depression among them was approximately 8.10% in the two cities. The samples of older participants from São Paulo and Manaus were different regarding sex (in São Paulo there were more women) and age (in Manaus there were more people aged 80 years and older). Older participants from São Paulo were more often single/separated, had a higher family income, and less comorbidities than those from Manaus. The proportion of older participants who correctly recognized the case in the vignette as a case of mental disorder were similar in São Paulo (40.5%) and Manaus (42.7%), *p* = 0.58 (Rao-Scott chi-square test).

**Table 1:** Characteristics of the Older Adults Registered in Primary Care Clinics, Stratified by City (%).

	São Paulo	Manaus	Total
Sex, female ^a^	61.0	57.9	59.9
Age categories ^a^			
60–69	58.3	53.0	56.3
70–79	31.8	31.7	31.7
≥ 80	9.90	15.3	12.0
Education (in years)			
0–4	78.0	71.9	75.7
≥ 5	22.0	28.1	24.3
Marital status ^a^			
Single/separated	52.7	44.9	49.8
Married/partnered	47.3	55.1	50.2
Family income (in R$) ^a^			
Up to 1000	33.3	49.0	39.6
1001–2000	43.1	40.7	42.1
≥2001	23.6	10.3	18.3
Physical morbidities, Yes ^a^	52.2	65.7	57.2
Physical limitations, Yes	9.30	10.3	9.70
Depression, PHQ-9 ≥ 10	8.20	7.60	8.10

^a^ Variable is associated with city at *p* ≤ .001 (Rao-Scott chi-square test).

**Table 2:** Characteristics of Primary Care Health Professionals Stratified by City [%, (*n*)].

	São Paulo	Manaus	Total
Sex, female	88.5 (201)	86.8 (210)	87.6 (411)
Age categories ^a^			
18–35 years	47.1 (107)	22.4 (54)	34.4 (161)
36–50 years	36.1 (82)	53.9 (130)	45.3 (212)
≥51 years	16.8 (38)	23.7 (57)	20.3 (95)
Marital status			
Married	64.9 (146)	62.0 (150)	63.4 (296)
Family income (in R$)			
1000–2000	36.4 (82)	44.2 (107)	40.5 (189)
2001–4000	33.3 (75)	24.0 (58)	28.5 (133)
≥4001	30.3 (68)	31.8 (77)	31.0 (145)
Self-perception of health			
Very good	19.5 (44)	28.5 (69)	24.2 (113)
Good	53.5 (121)	47.5 (115)	50.4 (236)
Not good	27.0 (61)	24.0 (58)	25.4 (119)
Depression, PHQ-9 ≥ 10	17.3 (39)	11.6 (28)	14.3 (67)
Health care worker type			
Community health worker	69.4 (154)	66.4 (152)	67.8 (306)
Nursing assistant	11.3 (25)	14.4 (33)	12.9 (58)
Nurse	12.1 (27)	9.60 (22)	10.9 (49)
Family physician	7.20 (16)	9.60 (22)	8.40 (38)
Works with patients with mental disorders, Yes ^a^	87.6 (197)	66.9 (162)	76.9 (359)

^a^ Variable is associated with city at *p* ≤ .001 (Chi-square test).

Since female community health workers represent the larger part of professionals in FHTs, females were also the majority in our sample (approximately 70% are community health workers and 87% of the sample are women). More than 65% of professionals from the two cities work with patients with mental disorders. Prevalence of depression in the two cities was high (in São Paulo, 17% and in Manaus, 12%). The only characteristics of health professionals that differed in São Paulo and Manaus were age and if they reported working with patients with mental disorders. Health professionals from São Paulo were younger and worked more frequently with patients with mental disorders than in Manaus. In São Paulo, health care worker type was associated with correct recognition of the case in the vignette as a case of mental disorder: community health worker (45%), nursing assistant (52%), nurse (70%), and family physician (94%), (*X*^2 =^ (3, n = 222) = 18.02, *p* < 0.001). In Manaus, this association was absent: community health worker (28%), nursing assistant (45%), nurse (41%), and family physician (36%), (*X*^2 =^ (3, n = 229) = 4.59, *p* = 0.20).

Table 3 presents the prevalence for the three domains of public stigma for older participants and health care professionals from São Paulo and Manaus.

**Table 3:** Prevalence of the Three Public Stigma Domains and its 95% CI among Older Adults and Health Care Professionals from São Paulo and Manaus.

	Older adults*		Health care professionals	
	São Paulo	Manaus	São Paulo	Manaus
Perceived negative reactions	31.5% (26.9–36.1)	35.4% (30.7–40.1)	31.7% (25.7–38.2)	43.4% (37.0–49.9)
Perceived discrimination	30.2% (24.4–36.1)	27.6% (23.1–32.1)	19.8% (14.8–.25.6)	30.2% (24.4–36.4)
Dangerousness	37.6% (30.7–44.4)	33.8% (29.1–38.6)	34.8% (28.6–41.4)	44.6% (38.3–51.1)

*Prevalence estimates adjusted considering the effect of cluster sampling.

Table 4 shows the unadjusted regression models for the three stigma domains among older participants. Perceived negative reactions showed statistically significant associations with depression and identification of the vignette as a case of mental disorder. Regarding perceived discrimination, statistical association was observed with physical morbidities, physical limitations, depression, and identification of the vignette as a case of mental disorder. Finally, dangerousness was statistically associated with physical limitations, and identification of the vignette as a case of mental disorder.

**Table 4:** Unadjusted Poisson Regression Models between Stigma Domains and Associated Factors in Older Adults Registered in Primary Care Clinics.

Variable	Perceived negative reactions	Perceived discrimination	Dangerousness
	RR (95% CI)	RR (95% CI)	RR (95% CI)
City (ref = São Paulo)			
Manaus	1.03 (0.98–1.08)	0.98 (0.93–1.04)	0.97 (0.92–1.03)
Sex Male	0.97 (0.92–1.02)	0.99 (0.94–1.05)	1.02 (0.98–1.07)
Age (years)	1.00 (0.99–1.00)	1.00 (0.99–1.00)	1.00 (0.99–1.00) ^b^
Education (ref = 0–4 years)			
≥5 years	1.02 (0.99–1.04)	1.00 (0.98–1.02)	1.01 (0.99–1.02)
Marital status (ref = married)			
Not married	1.04 (0.99–1.09) ^b^	1.00 (0.94–1.06)	0.97 (0.92–1.02)
Family income (ref = Up to R$1000)			
1001–2000	0.98 (0.93–1.03)	0.99 (0.93–1.06)	1.01 (0.95–1.06)
≥2001	0.98 (0.91–1.04)	1.01 (0.94–1.09)	1.06 (0.99–1.12)
Physical morbidities			
Yes	1.04 (0.98–1.10)	1.07 (1.03–1.12) ^a^	1.03 (0.98–1.08)
Physical limitation			
Yes	1.07 (0.99–1.16) ^b^	1.13 (1.03–1.23) ^a^	1.10 (1.02–1.20) ^a^
Depression (PHQ-9 ≥ 10)	1.12 (1.04–1.21) ^a^	1.17 (1.06–1.29) ^a^	0.98 (0.90–1.07)
Identification of the vignette as a case of mental disorder, Yes	1.09 (1.03–1.14) ^a^	1.12 (1.06–1.19) ^a^	1.15 (1.09–1.22) ^a^

RR = Risk Ratio; CI = Confidence Interval.

^a^ *p* < 0.05

^b^ 0.05 < *p* < 0.10

Note: Analyses were performed adjusting for city, as some of these variables were associated with it. As these analyses yielded the same results as the unadjusted ones, we present in the table results of the unadjusted regression models.

Table 5 shows the multivariate regression models for the three stigma domains among older participants. Older adults with depression and older adults who identified the vignette as a case of mental disorder were more likely to report that others would have perceived negative reactions towards the case in the vignette (RR = 1.12, 95% CI: 1.04–1.20, RR = 1.09, 95% CI: 1.03–1.14, respectively). Regarding perceived discrimination, older adults that presented physical morbidities were more likely to report that people would discriminate against the case in the vignette (RR = 1.06, 95% CI: 1.02–1.10). The same happened with those with physical limitations (RR = 1.10, 95% CI: 1.02–1.19), depression (RR = 1.15, 95% CI: 1.05–1.26) and those that identified the vignette as a case of mental disorder (RR = 1.12, 95% CI: 1.06–1.18). Participants with physical limitations had 10% more risk (RR = 1.10, 95% CI: 1.02–1.18) to perceive the case in the vignette as capable of being aggressive towards others. The same was observed with those that identified the vignette as a case of mental disorder (RR = 1.15, 95% CI: 1.09–1.22).

**Table 5:** Multivariate Models by Poisson Regression for the Three Stigma Domains among Older Adults.

Variable	RR_unadj_	RR_adj_	(95% CI)
Perceived negative reactions			
Depression (PHQ-9 ≥10), Yes	1.12	1.12	(1.04–1.20)
Identification of the vignette as a case of mental disorder, Yes	1.09	1.09	(1.03–1.14)
Perceived discrimination			
Physical morbidities, Yes	1.07	1.06	(1.02–1.10)
Physical limitations, Yes	1.13	1.10	(1.02–1.19)
Depression (PHQ-9 ≥10), Yes	1.17	1.15	(1.05–1.26)
Identification of the vignette as a case of mental disorder, Yes	1.12	1.12	(1.06–1.18)
Dangerousness			
Physical limitations, Yes	1.10	1.10	(1.02–1.18)
Identification of the vignette as a case of mental disorder, Yes	1.15	1.15	(1.09–1.22)

RR_unadj_: Unadjusted Relative Risk

RR_adj_: Adjusted Relative Risk

Table 6 shows the unadjusted regression models for the three primary stigma domains among primary health care professionals. The three stigma domains showed statistically significant associations with city. Perceived negative reactions and perceived discrimination were significantly associated with age. Dangerousness was statistically associated with sex.

**Table 6:** Unadjusted Poisson Regression Models between Stigma Domains and Associated Factors in Primary Health Care Professionals.

Variable	Perceived negative reactions	Perceived discrimination	Dangerousness
	RR (95% CI)	RR (95% CI)	RR (95% CI)
City (ref = São Paulo)			
Manaus	1.37 (1.08–1.74) ^a^	1.52 (1.10–2.10) ^a^	1.28 (1.02–1.61) ^a^
Sex			
Male	1.00 (0.70–1.43)	1.19 (0.78–1.84)	1.52 (1.17–1.96) ^a^
Age (years)	1.006 (1.00–1.01) ^a^	1.007 (1.00–1.01) ^a^	1.000 (0.99–1.00)
Marital status (ref = married)			
Not married	1.23 (0.97–1.55) ^b^	1.16 (0.84–1.60)	0.89 (0.70–1.13)
Family income (ref = R$ 1000–2000)			
2001–4000	0.75 (0.56–1.01) ^b^	0.97 (0.66–1.41)	0.93 (0.70–1.22)
≥4001	0.77 (0.58–1.02) ^b^	0.86 (0.59–1.26)	0.93 (0.72–1.22)
Self-perception of health (ref = Very good)			
Good	0.83 (0.63–1.09)	0.85 (0.58–1.24)	0.88 (0.67–1.17)
Not good	0.91 (0.66–1.24)	0.98 (0.64–1.49)	1.13 (0.84–1.52)
Depression (PHQ-9 ≥ 10)	0.76 (0.52–1.12)	0.74 (0.44–1.24)	0.92 (0.67–1.30)
Health care worker type (ref = Community health worker)			
Nursing assistant	1.06 (0.76–1.49)	0.95 (0.58–1.55)	0.91 (0.63–1.31)
Nurse	0.89 (0.59–1.34)	0.88 (0.50–1.53)	0.92 (0.62–1.36)
Family physician	0.95 (0.61–1.47)	1.13 (0.66–1.94)	1.25 (0.89–1.77)
Works with patients with mental disorders, Yes	1.06 (0.79–1.40)	0.91 (0.64–1.31)	1.09 (0.83–1.44)
Identification of the vignette as a case of mental disorder, Yes	0.95 (0.75–1.21)	1.27 (0.93–1.73)	1.23 (0.99–1.54) ^b^

RR = Risk Ratio; CI = Confidence Interval.

^a^ *p* < 0.05

^b^ 0.05 < *p* < 0.10

Table 7 shows the multivariate regression models for the three stigma domains among health care professionals. Health workers from Manaus compared with those in São Paulo were consistently more likely to report that people would have perceived negative reactions, discriminate and perceive the case in the vignette as capable of being aggressive towards others (34% (95% CI 1.06–1.70), 52% (95% CI 1.1–2.11), and 34% (1.07–1.68), respectively). A one year increase in age increases the RR of saying that others would have perceived negative reactions towards the case in the vignette in 0.5% (95% CI: 1.00–1.01). Men have 56% (95% CI 1.12–1.90) more risk while those who identified the vignette as a case of mental disorder have 28% more risk (95% CI 1.02–1.60) to perceive the case in the vignette as capable of being aggressive towards others.

**Table 7:** Multivariate Models by Poisson Regression for the Three Stigma Domains among Primary Health Care Professionals.

Variable	RR_unadj_	RR_adj_	(95% CI)
Perceived negative reactions			
City, Manaus	1.37	1.34	(1.06–1.70)
Age (years)	1.006	1.005	(1.00–1.01)
Perceived discrimination ^a^			
City, Manaus	1.52	1.52	(1.01–2.11)
Dangerousness			
City, Manaus	1.28	1.34	(1.07–1.68)
Sex, Male	1.52	1.46	(1.12–1.90)
Identification of the vignette as a case of mental disorder, Yes	1.23	1.28	(1.02–1.60)

RR_unadj_: Unadjusted Relative Risk

RR_adj_: Adjusted Relative Risk

^a^ For Perceived discrimination, the adjusted RR is the same as in the unadjusted model because city was the only variable that remained in the model.

## Discussion

This study examined the prevalence and factors associated with public stigma (perceived negative reactions, perceived discrimination and dangerousness) towards older adults with depression in two groups: older adults registered with PCCs and primary health care professionals in two large Brazilian cities, São Paulo and Manaus. The main finding is that the prevalence of public stigma is high both among older adults and among primary health care professionals. This study also found that older adults who correctly identified the vignette as a case of mental disorder presented an increased risk of 9% to 15% of reporting negative reactions, discrimination, and of saying that the person in the vignette could be potentially dangerous. Other characteristics of older adults associated with stigmatizing attitudes were presenting depressive symptoms, having any physical morbidity and/or limitation. Among older adults, the city they lived in (São Paulo and Manaus) was not associated with public stigma. On the other hand, primary health care professionals from Manaus consistently presented increased risk (34% to 52%) of reporting stigmatizing attitudes towards older people with depression as compared to those from São Paulo. Male primary care professionals and those who correctly identified the case in the vignette as a case of mental disorder reported higher risk of perceiving the case in the vignette as capable of committing a violent act against other people.

Perceived discrimination was the stigma domain with lower prevalence among older participants (30.2% in São Paulo and 27.6% in Manaus) and health care professionals (19.8% in São Paulo and 30.2% in Manaus), while dangerousness was the stigma domain with higher prevalence both among older participants (37.6% in São Paulo and 33.8% in Manaus) and health care professionals (34.8% in São Paulo and 44.6% in Manaus). Although agitation and irritability are sometimes symptoms of depression, the 70-year old case (Mary or John) depicted in the vignette clearly presents feelings of sadness, loss of interest in usual activities, changes in appetite and quality of sleep, and feelings of worthlessness. Hence, it is surprising that participants (older adults and health care professionals alike) from São Paulo and Manaus reported that the case in the vignette could potentially commit a violent act against other people. However, other recent studies have shown similar findings. For example, two previous studies carried out in different sociocultural contexts (Canada and Brazil) [15, 37] that assessed public stigma against depression with the same vignette as the one used in the present study but that depicted a much younger individual (30-years old) also found a high prevalence of perceived dangerousness. Compared to our study, a previous investigation in São Paulo[15] reported consistently higher prevalence in the three stigma domains; discrimination was the least prevalent (41%) and dangerousness the most prevalent (56%). Differences in prevalence rates may be because society perceives older adults as more harmless and fragile, therefore less likely to be perceived negatively, discriminated against and aggressive than younger people. Future studies could assess whether the age of the case in the vignette (besides its gender) influences perception of stigma. Our study indicated a prevailing social misconception that links mental illness to violence, although the literature shows that mental illness alone is not enough to make people more violent [38–40]. One possible explanation for discrimination being the least reported stigma domain in several investigations [15, 37, 41, 42] is that discrimination is the behavioral component of stigma and is possibly the one that is mostly susceptible to social desirability concerns. Behavior always manifests in public and is therefore under the scrutiny of others, whereas feelings and beliefs are more private.

Characteristics of older adults and primary health care professional associated with the three public stigma domains followed a different pattern. Among older adults, identifying the vignette as a case of mental disorder, and having depressive symptoms or physical morbidities or limitations increased the risk of perceived stigma. Primary health care professionals that worked in Manaus consistently showed more risk of having stigmatizing attitudes than those from São Paulo. Identification of the vignette as a case of mental disorder was only associated with perceived dangerousness, contrary to older participants for whom correct identification was associated with the three stigma domains.

Identifying mental illness is the first step to initiating adequate treatment. Unfortunately, one of the consequences of identifying a mental illness is labeling, which carries a negative connotation in many societies [15, 43, 44]. The use of labels that distinguishes “us” from “them" fosters stigma [45]. Stigma, in turn, hinders care seeking and treatment adherence [43]. Our findings are consistent with other studies showing that mental illness labels increase social distance and negative attitudes, [16, 17, 44, 46] especially among the general public. Identifying the case in the vignette as a case of mental disorder increased the risk of participants reporting that the individual in the case could be violent towards others. This result further supports the idea of a social misconception linking mental disorders to violence. Intervention targeting stigma against depression should start educating people about this misconception.

The proportion of older participants that correctly recognized mental disorder was similar in São Paulo and Manaus (approximately 40%). Among primary health care professionals, in São Paulo the more specialized the health care workers were, the more they correctly recognized mental disorder (range from 45% to 94%). In Manaus, health care worker position was not associated with accuracy. In São Paulo, the accuracy of community health workers (CHW) was similar to that of older adults. However, in Manaus older adults were more accurate than CHW, with their accuracy being similar to that of the more specialized professionals.

We investigated two Brazilian cities with very distinct cultural and socioeconomic backgrounds and considerably geographic distant (2,414 miles). Among older participants, socioeconomic and cultural context did not have an effect on public stigma towards older adults with depression. Thus, the similarities in our results provide evidence that among the Brazilian older population, public stigma against older adults with depression seems to be widespread. However, health care professionals from Manaus compared with those in São Paulo were more likely to report perceived negative reactions, perceived discrimination and dangerousness, and were less accurate in recognizing depression in the vignette. The Family Health Strategy is equally structured in the two cities, but the integration of mental health into primary care is still far from being adequate, with marked regional differences. In São Paulo, before the implementation of the FHS as the main primary health care public system, some primary care clinics had clinical psychologists and psychiatrists working with family physicians, and mental health outpatient services were available. In Manaus, the public mental health services have been and still are scarce, with only a few mental health specialists working in the public system. Importantly, in Manaus there are no psychiatry medical residency programs. These contextual differences may explain why professionals in Manaus were as accurate in identifying depression as older adults and why they showed more risk of saying that people with depression would be stigmatized than those from São Paulo.

Among older participants, having depression themselves was associated with perceived negative reactions and perceived discrimination, but not with dangerousness. These results are in line with previous studies showing that people with mental illness often internalize public stigma and believe they are less valued because of their mental illness [43, 47]. However, this study does not allow us to disentangle what is driving these effects: the participants’ self-stigma or the symptoms of depression *per se* which leads to more negative perceptions overall. Other individual characteristics, like physical morbidities and physical limitations increased perceived discrimination and dangerousness. Medically ill and physically limited individuals are target of stigma themselves. It may be that people that experience stigma themselves also recognize more stigmatizing attitudes in the society. Indeed, in our study older participants with these problems had increased perceptions that other people would discriminate and perceive the case in the vignette as being potentially dangerous. However, we did not assess the experience of being stigmatized due to medical morbidities and physical limitations therefore our interpretation regarding this finding is limited.

There are some limitations to this study. The vignette is an artificial situation that may not reflect true attitudes or behaviors. At the same time, social desirability might have threatened the validity of the assessment, as subjects might be embarrassed to report stigmatizing attitudes. However, we used a validated measure that is widely employed in studies investigating stigma. Another limitation is that this is a cross-sectional survey, thus it does not measure changes in the level of stigma over time and therefore does not indicate the direction of the associations. Lastly, this study was carried out with older adults registered with PCCs with family health teams, and primary care professionals working in PCCs. Since the sample was recruited from a primary care setting, our findings may not be generalizable to the small proportion of older adults that use the private health sector as well as health care workers that work only in private practice. However, the FHS framework covers approximately 60% of the Brazilian population.

In summary, our study confirms that stigma against the elderly with depression by both the public and health care community in Brazil is common. One key factor associated with stigmatizing beliefs is the labeling of depression as a mental illness. There have been several studies that have shown that people with mental illness receive fewer medical services than those not labeled as mentally ill [48, 49]. It is important to educate primary health care providers and the general public in Brazil on stigma related to mental illness in order to reduce barriers for older individuals with depression to receiving appropriate medical and mental health care.

### Ethics

All study procedures were approved by the Research Ethics Committee at the Faculty of Medicine at the University of São Paulo, Municipal Secretary of Health of São Paulo, the University of Amazonas State, and the Municipal Secretary of Health of Manaus.

## Supporting Information

S1 Dataset São Paulo-Manaus Study–Elderly Data. (DTA)

S2 Dataset São Paulo-Manaus Study–Health Care Professionals Data. (DTA)
